# Effects of the COVID-19 Pandemic and Telehealth on Antenatal Screening and Services, Including for Mental Health and Domestic Violence: An Australian Mixed-Methods Study

**DOI:** 10.3389/fgwh.2022.819953

**Published:** 2022-06-22

**Authors:** Amanda Henry, Jennifer Yang, Sarah Grattan, Lynne Roberts, Anne Lainchbury, Janani Shanthosh, Patricia Cullen, Louise Everitt

**Affiliations:** ^1^Discipline of Women's Health, School of Clinical Medicine, UNSW Medicine and Health, University of New South Wales (NSW), Sydney, NSW, Australia; ^2^Department of Women's and Children's Health, St George Hospital, Sydney, NSW, Australia; ^3^The George Institute for Global Health, UNSW Medicine and Health, Sydney, NSW, Australia; ^4^St George and Sutherland Clinical School, UNSW Medicine and Health, University of New South Wales, Sydney, NSW, Australia; ^5^Royal Hospital for Women, Randwick, NSW, Australia; ^6^Australian Human Rights Institute, UNSW Sydney, Kensington, NSW, Australia; ^7^School of Population Health, UNSW Sydney, Kensington, NSW, Australia; ^8^Ngarruwan Ngadju, First Peoples Health and Wellbeing Research Centre, Australian Health Services Research Institute, University of Wollongong, Wollongong, NSW, Australia; ^9^School of Nursing and Midwifery, Western Sydney University, Penrith, NSW, Australia

**Keywords:** pregnancy, mental health, domestic and family violence, COVID-19, telehealth, pregnancy care

## Abstract

**Introduction:**

Australian antenatal care includes specific screening and service provision for domestic and family violence (DFV) and mental health. However, the COVID-19 pandemic resulted in major care changes, including greatly expanded telehealth. Given difficulties in a safe assessment and management of disclosures *via* telehealth, DFV and mental health service provision might be substantially impacted. This study therefore aimed to assess COVID-19 effects on DFV and mental health screening, as well as broader service provision from the perspective of local maternity service providers.

**Methods:**

Mixed-methods study of staff surveys and interviews of staff directly involved in pregnancy care (doctors, midwives, and allied health) in three Sydney (Australia) maternity units, from October 2020 to March 2021. Surveys and interviews interrogated perceived effects of the COVID-19 pandemic on delivery (ensuring required services occurred), timeliness, and quality of (a) overall maternity care and (b) DFV and mental health screening and care; and also advantages and disadvantages of telehealth. Surveys were descriptively analyzed. Interviews were conducted online, recorded, and transcribed verbatim prior to thematic analysis.

**Results:**

In total, 17 interviews were conducted and 109 survey responses were received. Breakdown of survey respondents was 67% of midwives, 21% of doctors, and 10% of allied health. Over half of survey respondents felt the pandemic had a negative effect on delivery, timeliness, and quality of overall pregnancy care, and DFV and mental health screening and management. Perceived telehealth positives included convenience for women (73%) and reducing women's travel times (69%). Negative features included no physical examination (90%), difficulty regarding non-verbal cues (84%), difficulty if interpreter required (71%), and unsure if safe to ask some questions (62%). About 50% felt telehealth should continue post-pandemic, but for <25% of visits. Those perceived suitable for telehealth were low-risk and multiparous women, whereas those unsuited were high-risk pregnancy, non-English speaking, and/or mental health/psychosocial/DFV concerns. “Change to delivery of care” was the central interview theme, with subthemes of impact on mental health/DFV screening, telehealth (both positive and negative), staff impact (e.g., continuity of care disruption), and perceived impact on women and partners.

**Discussion:**

While telehealth may have an ongoing, post-pandemic role in Australian maternity care, staff believe that this should be limited in scope, mostly for low-risk pregnancies. Women with high risk due to physical health or mental health, DFV, and/or other social concerns were considered unsuited to telehealth.

## Introduction

The COVID-19 pandemic has dramatically impacted communities globally across multiple areas of life, including healthcare and access to routine care such as pregnancy care. In Australia during 2020 (first and second waves), the period this study focuses on, burden of disease secondary to COVID-19 was low on an international scale, with only 18 cases of COVID-19 in pregnancy reported in New South Wales (the study setting) in the first wave (1). However, as has been reported around the world (2), routine healthcare including antenatal care was greatly impacted in Australia, with Medicare billing for face-to-face antenatal care services declining 15% in second quarter of 2020 compared to 2019 (3).

The final impact of social isolation, lockdowns, and various restrictions to prevent the spread of COVID-19 is also still to be fully realized. These measures, as well as associated stressors such as unemployment and schooling from home, are expected to dramatically increase women's risk of domestic and family violence (DFV), (4–6) the single greatest cause of death, ill health, and disability in reproductive-age Australian women (7). Pregnant women are a vulnerable group regarding DFV, with an estimated 187,800 Australian women who have experienced violence by a current partner pregnant at some stages during the relationship and 18% of these women experiencing violence during their pregnancy (8). As well as seeing women who are actively experiencing violence during their pregnancy, maternity care providers also see women who have previously experienced intimate partner violence and who are still living with the ongoing consequences for themselves and their children.

Mental health presentations, including depression and anxiety, are also very common both during and after pregnancy. Australian and overseas studies report antenatal depression rates of approximately 10% and anxiety prevalence up to 20% in late pregnancy (9).

In general, pregnancy care is one of the times in a woman's contact with Australian healthcare services where psychosocial screening, including DFV and mental health screening, is routine and has systems in place to provide appropriate support. This care is evidence-based and acceptable to women, allowing for risk assessment, safety planning, appropriate follow-up, and potentially decreasing post-traumatic stress disorder (PTSD) and perinatal depression as well as improving mother–child interactions (9–11).

To address DFV during the COVID-19 pandemic, the Australian government increased funding for telehealth and online support services. However, telehealth (meaning either telephone or videoconferencing/online consultation, referred to collectively as “telehealth” throughout this article) relies on women being able to speak in private and access referral pathways into community-based frontline services. Use of telehealth for the “booking-in” pregnancy visit, which is usually one of the time points for routine psychosocial screening in Australia, potentially particularly affected DFV and mental health screening. Women may not be as comfortable to disclose these issues when not in a face-to-face setting and/or may not be in a safe and private setting when booking-in from home. Accordingly, current New South Wales (NSW) Health guidelines recommend deferring DFV screening until the first face-to-face visit (12), which may be as late as 28-week gestation, delaying screening and management of any disclosures.

Although several studies have examined the overall impact of the pandemic on perinatal mental health and/or DFV, there is very limited focus on the impact of maternity systems change and its impact on screening. A rapid evidence review on women's mental health during pregnancy in the pandemic included 17 studies and found that anxiety and depressive symptoms ranged from 29.6 to 72%, more than doubled during the pandemic (12, 13). Regarding violence in pregnancy, both an Iranian and Canadian study found high levels of intimate partner violence in the early months of the pandemic (14, 15), however, neither had pre-pandemic controls for comparison.

Regarding COVID-19's impact on maternity care provision generally, a 2021 global scoping review reported that prenatal care visits decreased, healthcare infrastructure was strained, and potentially harmful policies such as increasing time between antenatal visits were instituted (16). While the replacement of in-person visits with telehealth saw some benefits, such as increasing access to care and therefore appointment attendance rates, reducing wait times, and avoiding exposure to COVID-19, barriers identified included technical difficulties and privacy concerns. In general, management of workflow, and convenience for both staff and women, having some pregnancy care visits *via* telehealth rather than face-to-face may work very well and is likely to continue post-pandemic. It is therefore very important for maternity care services to also understand the limitations of telehealth for antenatal care, and in particular, the effect on those with complex psychosocial needs who are often experiencing broader inequities, to plan appropriate care as Australia emerges from the COVID-19 pandemic.

The aim of this study was therefore to examine, from the perspective of maternity staff, the effect of the COVID-19 pandemic on provision of maternity care in the South-Eastern Sydney Local Health District (SESLHD), Australia, particularly on the identification and management of mental health, psychosocial issues, and domestic and family violence. It also explored the implications of telehealth in antenatal care and its application moving forward.

## Materials and Methods

A mixed-methods study comprising surveys and interviews was conducted among maternity staff of SESLHD, New South Wales, Australia. In Australia, the main maternity care clinicians are registered midwives (who may or may not also have a nursing qualification), doctors, and allied health staff including social workers, physiotherapists, genetic counselors, and Aboriginal health workers. Staff were eligible if they were currently registered and practicing midwives, obstetric medical staff, or allied health staff working in the obstetric/maternity units of St George Hospital (SGH), the Royal Hospital for Women (RHW) or Sutherland Hospital (TSH), the three hospitals in SESLHD which provide pregnancy and birth care. Staff were only eligible if they had worked in SESLHD Maternity during 2019 and 2020, to allow for comparison of experiences pre-pandemic and during pandemic. For context, the three hospitals have different service capabilities and patient populations: RHW is the area's tertiary maternity referral center, performing ~3,800 births/year, with full neonatal intensive care facilities and co-located with neonatal surgical facilities and adult intensive care, and located in a high sociodemographic status area. SGH performs ~2,400 births/year, has a special care nursery (births 32 weeks and above) and full adult intensive care facilities but not neonatal intensive care, and is situated in a highly diverse sociodemographic area, with approximately half of its maternity population born overseas in a country where English is not the first language. TSH is a smaller unit, performing ~1,200 births/year, 34 weeks and above and transferring out women with major medical conditions such as preeclampsia and type 1 diabetes, and has a majority Caucasian/Australian born catchment area.

### Staff Survey

An anonymous online survey (Supplementary Material 1) was distributed *via* staff's email to all midwifery, obstetric medical, and allied health staff providing frontline maternity care services in SESLHD maternity facilities (RHW, SGH, TSH)—estimated to be approximately 500 staff in total. The survey included:

- demographic questions (hospital, age range, type of healthcare professional, years of experience range)
- questions about perception of the *overall* impact of the COVID-19 pandemic on delivery, timeliness, and quality of pregnancy care
- questions about pandemic impact on delivery, timeliness, and quality of (a) domestic and family violence screening and care (b) mental health screening and care
- questions about perception of telehealth (positives, negatives, women suited and not suited for telehealth, group/antenatal education impact of telehealth)

All staff were invited to participate and emailed the survey link, up to three times between November 2020 and January 2021. As not all frontline staff regularly access their NSW Health emails, flyers regarding the study were also posted in maternity staff common areas (with QR code to link to survey), and an in-service about the study given at each participating hospital to answer questions about the study and provide maternity staff with details of how to participate if they wished to do so. Completion of the survey was taken as consent to participate.

### Interviews

Semi-structured interviews (Supplementary Material 2) were conducted with maternity healthcare staff to explore in detail their perceptions of the impact of the COVID-19 pandemic on provision of antenatal care and maternity services, with a focus on their impressions of impacts on mental health/psychosocial screening and DFV screening. Interviews were conducted after the survey distribution period; however, the survey and interview guide were developed in parallel prior to study commencement. To streamline recruitment for staff interviews, the final question of the survey asked whether staff would be interested in participating in an interview. If so, they were asked to enter contact details into the survey (with response to this question separated from response to other aspects of survey to maintain survey participant anonymity). If that did not yield an appropriate cohort of participants with representation from each hospital and each discipline, purposive sampling of initially under-represented staff occurred, *via* sending to the SESLHD emails of under-represented maternity clinician types and/or under-represented hospital maternity staff, an invitation to participate in interviews.

Potential participants were provided prior to interview with information regarding the purpose of the interviews, that participation was voluntary, and that their identity would be protected through de-identification during the transcription process and in reporting of study findings (Supplementary Material 3). Interviews were planned to be no longer than 1 h and to take place online (*via* zoom or Skype). Interviews were performed by study staff (SG) with no direct employment links within SESLHD/not a work colleague of the interviewees, to minimize participation or response bias due to the interviewer having a pre-existing work relationship with the interviewees. With participant consent, the interviews were audio-recorded to allow for the ease of later transcription and coding. Interviews continued until there was representation of each of the participating hospitals and maternity care professionals (midwives, doctors, and allied health), and saturation of themes occurred.

### Data Analysis

1) Surveys: Data were downloaded to SPSS (IBM SPSS Statistics for Windows, V27, Armonk, NY) and analyzed and reported using descriptive statistics (number and percentage) for closed answer questions. Where respondent subgroup size allowed (i.e., at least five in each subgroup, so that there would be no possibility of identifying individuals), then responses to questions about overall pregnancy care and specific DFV and mental health screening were analyzed by (a) hospital of practice, (b) type of maternity healthcare professional, and (c) length of time working in maternity services, with subgroup responses compared using chi-squared testing. The open-ended questions were analyzed and reported thematically.

2) Interviews: Transcripts were produced for each individual recording and initially screened by the interviewer (SG) to remove any potentially identifying details before sharing with senior authors AH and LE. Data were analyzed using the thematic approach outlined by Braun and Clarke (17) consisting of deep familiarization with the data; searching for themes; reviewing, defining and naming the themes; and finalizing the analysis. SG, AH and LE performed the analysis, each reviewing transcripts and discussing themes and subthemes until agreement was reached to validate the findings. These are illustrated by typical excerpts from participants, identified only by professional grouping (as professional grouping and hospital might inadvertently identify participants).

### Ethical Approval

The studies involving human participants were reviewed and approved by South-Eastern Sydney Local Health District Human Research Ethics Committee (Ref: ETH01518/2020). The participants provided their written informed consent to participate in this study (interviews), while for anonymous survey participation, the completion of survey was taken as evidence of consent to participate.

## Results

### Surveys

A total of one hundred and nine survey responses were received (~20% of estimated total SESLHD maternity staff): 75 from midwives (69%), 23 from medical staff (21%), and 11 from allied health (10%). As shown in Table 1, respondents were overwhelmingly female-identifying (97%) in keeping with the overall maternity care workforce, approximately half were aged 44 and under and half 45 or older, and TSH was slightly under-represented (9% of respondents) in comparison with its proportion of SESLHD births (~16%).

**Table 1:** Demographic characteristics of survey respondents.

**Total survey respondents**	***N* (%), total *N =* 109**
**Primary discipline**
**Midwifery**	75 (69)
Antenatal care	17 (16)
Postnatal care	9 (8)
Intrapartum care	10 (9)
Midwifery Group Practice	11 (10)
CMC, CMS or CME	11 (10)
Management	4 (4)
All areas	11 (10)
Midwife, prefer not to say area	1 (1)
**Medical**	23 (21)
Obstetrician, work predominantly public	4 (4)
Obstetrician, work equal public and private	3 (3)
Obstetrician, work predominantly private	2 (2)
Obstetric Registrar/Resident	14 (13)
**Allied Health**	11 (10)
Social work	1 (1)
Physiotherapy	4 (4)
Other	6 (6)
**Sex**
Female	106
Male	2
Non-binary	1
**Age (years)**
<25	2 (2)
25–34	33 (30)
35–44	22 (20)
45–54	23 (21)
55 and older	27 (25)
Prefer not to say	2 (2)
**Years of Experience**
5 or less	25 (23)
6–10	26 (24)
Between 11 and 15	13 (12)
16 or more	43 (39)
Prefer not to say	1 (1)
**My primary affiliated public hospital:**
Royal Hospital for Women	62 (57)
St George Hospital	37 (34)
Sutherland Hospital	10 (9)

Table 2 shows the survey respondents' perception of pandemic impact on (1) delivery and (2) timeliness of overall pregnancy care, mental health screening, and domestic and family violence screening, as well as impact on (3) quality of overall, mental health, and DFV screening and care. The proportions who viewed the pandemic as having a somewhat negative or extremely negative impact were over 50% for all categories. However, more staff rated pandemic effects on delivery (*p* = 0.02) and timeliness (*p* = 0.004) of DFV screening (but not quality of care) as *extremely* negative vs. effects on overall pregnancy care.

**Table 2:** Staff perceptions of pandemic impact on overall care and on psychosocial screening^#^.

**Impact on delivery**	**Overall pregnancy care *N* (%)**	**Mental health screening *N* (%)**	**DFV screening *N* (%)**	***P*-value overall vs. mental health**	***P*-value overall vs. DFV**
Extremely negative	10 (9)	16 (15)	22 (20)	0.21	**0.02**
Somewhat negative	56 (51)	46 (42)	40 (37)	0.18	**0.03**
Neutral	29 (27)	19 (17)	22 (20)	0.39	0.26
Somewhat positive	8 (7)	10 (9)	4 (4)	0.62	0.24
Extremely positive	3 (3)	1 (1)	1 (1)	0.62	0.62
Unsure	3 (3)	17 (16)	20 (18)	**0.001**	**<0.001**
**Impact on timeliness**	**Overall pregnancy care** ***N*** **(%)**	**Mental health screening** ***N*** **(%)**	**DFV screening** ***N*** **(%)**	***P*** **-value overall vs. mental health**	***P*** **-value overall vs. DFV**
Extremely negative	8 (7)	17 (16)	23 (21)	0.06	**0.004**
Somewhat negative	59 (54)	43 (39)	41 (38)	**0.03**	**0.01**
Neutral	24 (22)	24 (22)	21 (19)	1.0	0.62
Somewhat positive	6 (6)	7 (6)	4 (4)	0.76	0.52
Extremely positive	0 (0)	1 (1)	1 (1)	1.0	1.0
Unsure	12 (11)	16 (15)	19 (17)	0.42	0.18
**Impact on quality**	**Overall pregnancy care** ***N*** **(%)**	**Mental health screening and care** ***N*** **(%)**	**DFV screening and care** ***N*** **(%)**	***P*** **-value overall vs. mental health**	***P*** **-value overall vs. DFV**
Extremely negative	11 (10)	6 (6)	12 (11)	0.21	0.83
Somewhat negative	60 (55)	51 (47)	44 (40)	0.22	**0.03**
Neutral	25 (23)	26 (24)	26 (24)	0.87	0.87
Somewhat positive	7 (6)	9 (8)	5 (5)	0.61	0.55
Extremely positive	2 (2)	2 (2)	3 (3)	1.0	1.0
Unsure	4 (4)	15 (14)	19 (17)	**0.008**	**0.001**

*^#^See Supplementary Material 1 questionnaire for examples/prompts given to staff regarding delivery, timeliness and quality of care. Bold values means statistically significant result (p <0.05)*.

Regarding subgroup perceptions (Table 3), several statistically significant differences were noted according to hospital site. Staff at SGH (the hospital with the highest diversity population) overall had more negative perceptions about pandemic impact, particularly on the delivery, timeliness, and quality of DFV and mental health care, as well as quality (but not timeliness or delivery) of overall pregnancy care. Few significant differences were noted between the professions (midwifery, medical, and allied health), apart from a higher proportion of allied health and medical staff than midwifery staff being “not sure” about delivery, timeliness, and quality of overall care and mental health and DFV screening and care. There were no major differences in staff perceptions by years of experience.

**Table 3:** Staff with a negative perception of COVID-19 impact by hospital.

**Very or somewhat negative impact on:**	Royal Hospital for Women*N (%)	St George Hospital**N (%)	Sutherland Hospital***N (%)	***P*-value**
*Delivery* of Overall Pregnancy Care	35/62 (56)	26/37 (70)	5/10 (50)	0.31
*Delivery* of Mental Health screening	29/62 (47)	29/37 (78)	4/10 (40)	0.005
*Delivery* of DFV screening	28/62 (45)	29/37 (78)	5/10 (50)	0.008
*Timeliness* of Overall Pregnancy Care	35/62 (56)	26/37 (70)	6/10 (60)	0.34
*Timeliness* of Mental Health screening	28/62 (45)	28/37 (76)	4/10 (40)	0.008
*Timeliness* of DFV screening	29/62 (47)	30/37 (81)	5/10 (50)	0.003
*Quality* of Overall Pregnancy Care	33/62 (53)	33/37 (89)	5/10 (50)	0.001
*Quality* of Mental Health screening	26/62 (42)	25/37 (68)	6/10 (60)	0.047
*Quality* of DFV screening	25/62 (40)	27/37 (73)	4/10 (40)	0.005

^*^Royal Hospital for Women, Area's tertiary hospital, full maternity and neonatal facilities, high sociodemographic status area overall.

^**^St George Hospital, Full maternity facilities, neonatal >32 weeks facilities, high sociodemographic diversity with approximately half of maternity population born overseas in predominantly non-English speaking country.

^***^Sutherland Hospital, Lower risk unit, majority Caucasian Australian population.

*DFV, Domestic and family violence*.

Regarding telehealth, as shown in Table 4, there was a major shift in its use for pregnancy care. Over 75% of respondents reported that pre-pandemic, <10% of visits were by telehealth. During the pandemic, this shifted to only 10% stating no telehealth, with the majority (52%) stating over 10% of visits occurred by telehealth. Most respondents nominated two or more telehealth advantages, most frequently convenience for the woman (73%), reducing longer travel times for some women (69%), and reducing clinic overcrowding (62%). However, more respondents nominated multiple negative features, including inability to do physical examination (90%), difficulty picking-up non-verbal cues (84%), difficult if interpreter required (71%), and unsure if safe to ask some questions (62%: majority noting DFV questions as unsure if safe to ask). A total of 29% felt telehealth increased inequity in pregnancy care. Regarding post-pandemic telehealth, 56% felt telehealth should definitely or probably be used for some aspects of pregnancy care, “sometimes” (10–25% of visits). Staff also felt that there were groups of women particularly suited or not suited to telehealth. Of the 64 respondents who nominated those particularly suited to telehealth, 55% nominated low-risk women, 52% multiparous women, and 34% those living further from hospital. Of 81 respondents nominating groups not suited to having any visits by telehealth, 65% were concerned regarding women with high-risk pregnancy/medical co-morbidities, 53% for non-English speaking women, and 25, 19, and 26% for women with mental health presentations, DFV issues, and other psychosocial issues, respectively.

**Table 4:** Telehealth frequency pre- and during pandemic.

***N* (%) telehealth antenatal/pregnancy care visits**	**Pre-pandemic**	**During pandemic**	***P*-value**
None	73 (67)	11 (10)	**<0.001**
Occasional (<10%)	12 (11)	22 (20)	0.06
Sometimes (10–25%)	1 (1)	28 (26)	**<0.001**
Often (26–50%)	4 (4)	22 (20)	**<0.001**
Majority (over 50%)	3 (3)	7 (6)	0.20
Not sure/couldn't say	16 (15)	19 (17)	0.58

*Bold values means statistically significant result (p <0.05)*.

### Interviews

**Figure 1:**
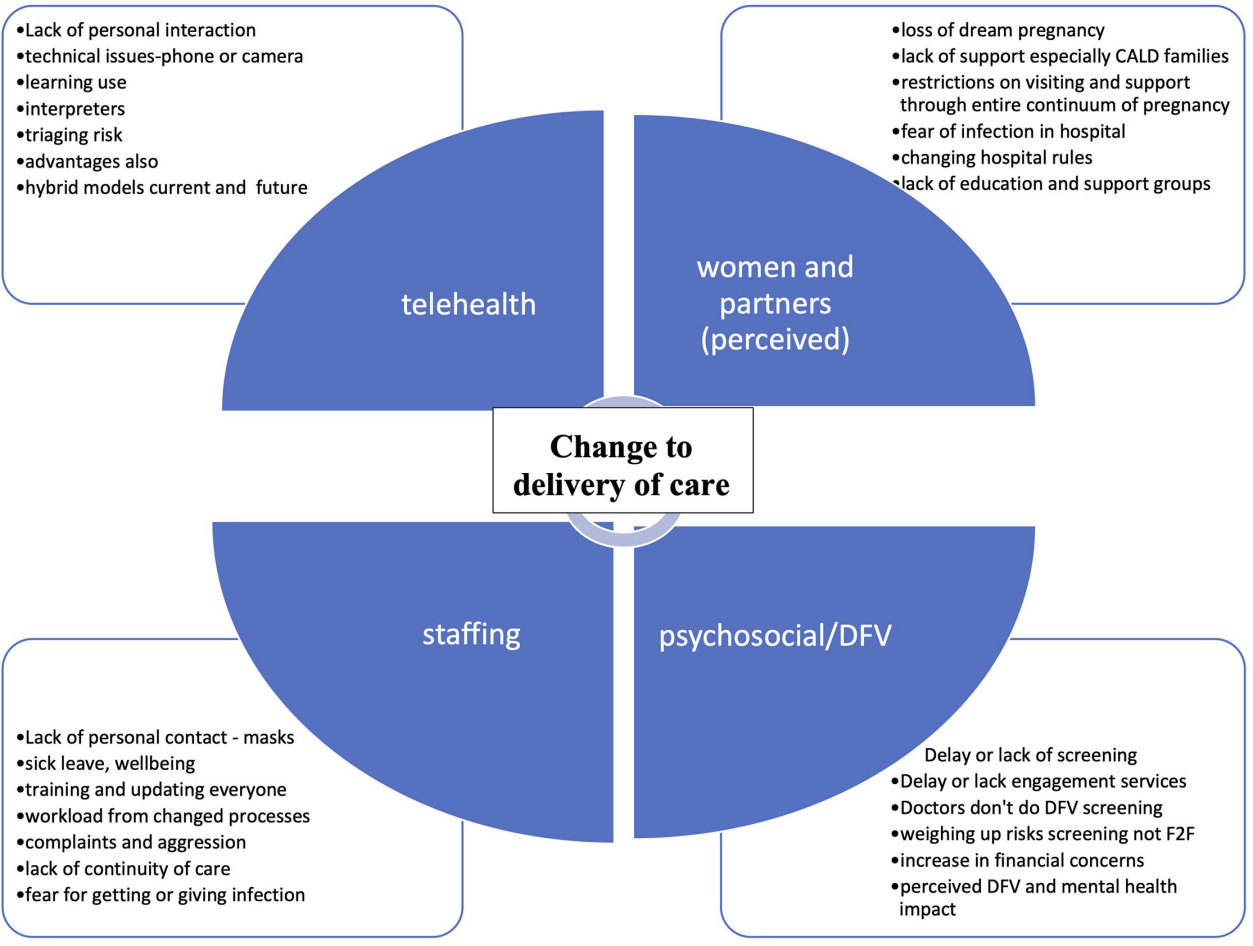
Themes from interviews. DFV, domestic and family violence; F2F, face-to-face; CALD, culturally and linguistically diverse.

A total of 17 interviews were conducted (10 midwives, 3 medical staff, and 4 allied health staff). Targeted sampling *via* email invitation was required to achieve an appropriate sample due to insufficient staff indicating an interest in interview at the time of survey completion. Sufficient midwifery staff sampling was achieved with the first targeted email invitations, while a second round of email invitations was required to achieve sufficient allied health and medical staff. After the first 14 interviews, no further new thematic areas were identified in the subsequent three interviews and therefore interviews ceased. As shown in Figure 1, the central theme from the interviews was the changes to delivery in care resulting from the pandemic. The major issues arising from this theme were telehealth, psychosocial/DFV considerations, perceived effects on women and partners, and effects on staff. Although positive as well as negative aspects of changes to delivery in care were nominated by staff, there was an overall sense that women's health was being sacrificed on behalf of the community, with loss of usual emphasis on woman-centered care (18). This was summarized by one midwife:

“The changes we needed to put in place were not woman-centered at all…it was governed by the needs of the greater community with COVID and the changes that we had to make.” Midwife 108

#### Change to Delivery of Care—Telehealth

All interviewees noted the major change to delivery of care wrought by the shift away from face-to-face visits and toward telehealth. For most, telehealth was seen as an inferior albeit necessary substitute noting that lack of face-to-face appointments impacted the ability to communicate and care for women. This midwife describes what the women were saying:

“We found that women were reporting to us that they didn't feel cared for…until they had their first face-to-face appointment, and then they finally felt they were pregnant and they were being looked after.” Midwife 105

One of the allied health interviewees described the difficulty of engaging on the phone:

“Talking to women is so much…not just easier, but a better way to develop that rapport with someone…Talking face-to-face to somebody over the phone that doesn't really want to talk to us…it's very hard.” Allied health 114

For some, this impact on effective communication with women was further impacted by technical and equipment issues. Interviewees noted the lack of appropriate equipment, meant that they could not actually work properly:

“I think it could have been good, if this organization was invested in the equipment…It took me four months to get a computer that was a laptop, and I still haven't been able to crack how to get those two apps on my desktop…so I still cannot work remotely.” Midwife 102

“I didn't even have the camera on my screen until like after the events [first wave 2020].” Allied Health 114

Some recognized the potential advantages of telehealth and, however, acknowledged that it may not be appropriate for the pregnant population. One doctor described how being unable to perform physical examinations would not provide appropriate care:

“I think with the provision of a lot of telehealth services…good for cover in other areas of medicine…but antenatal care was very difficult …because our routine check-up of a fetal heart or blood pressure can't be done over telehealth…so I don't feel as though the telehealth was able to sufficiently care for these women.” Medical 107.

A midwife also felt the outcomes of care with telehealth could actually be poorer in pregnancy:

“I understand telehealth is a fantastic innovation…I can see where it has amazing value, but I think in the maternity care context, particularly in the urban setting and…high risk pregnancy…if they engage with antenatal care early, the outcomes are fantastic…whereas with telehealth it isn't the same.” Midwife 109

However, for postnatal care where the woman with her baby could be seen telehealth had advantages:

“they set the whole scene up where we can see the baby and we can watch breastfeeds…we would never have been able to achieve this without telehealth.” Midwife 108

Telehealth was also perceived as offering women an option of care and convenience, in particular for women living further away from hospital (one maternity unit clients not uncommonly live over 30-min drive from the hospital):

“We do a telehealth service for women who are just out of our boundary…you can reach more women or you can make things a bit more accessible to them.” Midwife 101

Post-pandemic, some voiced that a hybrid model of face-to-face and telehealth would likely be adopted for some women; however, the challenge was to ensure the technology and establish criteria for who would be suitable:

“it [telehealth] would work for some and not work for others…going with the hybrid model antenatally, some women are much, much happier doing the video calls…we've seen it work so we can offer that but…we realized actually that some women still need a lot more visits and a lot more care…we have gone back to kind of normal visits for them.” Midwife 104

#### Changes to Delivery of Care—Psychosocial and Domestic and Family Violence Screening and Care

In line with the survey results, most interview participants viewed the combination of booking visits occurring by telehealth, and face-to-face visits occurring later in gestation (and less frequently), as particularly detrimental to timely screening and care for psychosocial and DFV issues. Some women missed screening altogether, as noted by one midwife:

“..very difficult to gain the intimate information over the phone…and because we dropped some of the appointments…sometimes we didn't get to ask these questions through the whole pregnancy. I have had a few women who went home without ever being asked.” Midwife 110

Some staff initially planned to screen *via* telehealth, so that identification of issues would not be delayed, but safety concerns precluded this:

“Because the bookings were being done on telehealth, we found it very difficult to do the DV [domestic violence] screen. In fact, we didn't do the DV screen because one day we were doing a booking and we asked the woman is she was on her own she said ‘Yes' and then we did the DV screen and the next thing her husband spoke…So, we then decided we had to stop.” Midwife 109

Re-instituting face-to-face visits occurred because the risks of COVID-19 were outweighed by the risks of poor psychosocial care, and staff recognized the inequity of failing to adequately provide screening and care:

“After a bit we just said, ‘Well, actually, weighing up the risks, the risk of somebody being harmed by domestic violence was higher than the risk of them actually catching COVID if they came to our hospital, and so we just started doing face-to-face appointments again…It's our responsibility. If we're going to screen and ask clients for information about their backgrounds, their experiences…we have to do something about it.” Allied Health 112

Many participants also recognized delaying the initial psychosocial screening *via* telehealth, even when the woman was subsequently face-to-face, meant screening may not have been done, as women were often seen by a doctor and they are not used to doing the psychosocial/DFV screen. One midwife explained:

“we might not see them until 31 weeks…GPs [general practitioners] would be seeing them in between but GPs don't usually ask these questions, and [antenatal clinic] doctors don't always either.” Midwife 104

Doctors also recognized that it was not something they usually did:

“very occasionally in the doctor's clinic…the midwives will put a little sign on the file to say please complete her EDS [Edinburgh Perinatal Depression Scale], or repeat her EDS if it was sort of borderline at the previous visit or whatever, but I have to say that's not something we routinely do, and probably not something we do all that well, necessarily.” Medical 115

For the women, this had flow-on effects of delayed screening becoming delayed referral to services and so delay or lack of care in the window of opportunity that pregnancy provides:

“the maternity screening wasn't happening as early…because they were doing some phone work…then for that reason they weren't getting as many disclosures in relation to domestic violence, which then postponed our referrals….didn't give us as much time to deal with those cases.” Allied Health 114

However, some did note an unexpected positive of restrictions, particularly partners not being allowed to come in to antenatal visits, regarding psychosocial screening:

“Sometimes it's difficult to get a woman by herself if she's…in a volatile relationship. And the fact that the hospital's enforcing it means…you have that protected time with women.” Midwife 101

Another midwife felt increased disclosures occurred:

“The women were here without their husbands so we've had a lot more disclosure.” Midwife 110

Overall, the changed mode of appointments to telehealth, delay in asking psychosocial screening questions, and reduced face-to-face visits were perceived as impacting the care women could receive. In some cases, no or lack of screening meant opportunities were missed to provide a safe environment for women to choose to disclose DFV or psychosocial concerns and be offered appropriate supports.

#### Change to Delivery of Care-Perceived Effect on Women and Partners

Maternity care staff noted both practical effects of delivery of care changes and also perceived impacts on the emotional well-being of women and partners (which were in turn seen to be related to overall pandemic effects). Practical impacts on women and partners were both positive and negative from a staff perspective. An unexpected positive of the pandemic care changes was regarding postnatal care: rules not allowing visitors apart from the partner on postnatal ward meant that women got more opportunity for assistance with breastfeeding and postnatal recovery, the midwives noted:

“It is nice to not have 10 visitors in during the day so that you can do that education, get that breastfeeding embedded…sometimes people can get lost in a sea of visitors….or they don't feel comfortable having those conversations with the midwives about perineal care…because they've got a room full of visitors and feel like they have to entertain and pass the baby around and that kind of thing.” Midwife 101

“Postnatally, I think the women have recovered a lot better because it's gone back to the old days, 40 days of rest, because no one can visit them.” Midwife 108

However, restrictions antenatally, including education all switching to online, were seen as detrimental to preparation for labor, birth, and parenting. One doctor described this negative impact:

“Some things like education groups stopped running...which I think was a great shame…the maternity tours stopped running, so women, I think felt…a little bit more alienated from you know, the process of what was going to happen when they came into labor.” Medical 115

While one of the midwives noticed a change this had on fathers:

“I noticed the fathers seemed to be traumatized more about the birth experience than they used to be…the only thing I could put it down to was the fathers weren't being permitted to come into the hospital for antenatal classes.” Midwife 102

One midwife summarized the effects of care change positives and negatives as:

“So, postnatally, definitely better. Antenatally, I think we'll see repercussions down the track.” Midwife 108

Although some impacts on emotional well-being of changes to delivery of care were difficult to separate out from the general effects of the pandemic, there was a strong sense from staff of the negative impact of care delays, restrictions, and reduced face-to-face opportunities. These were felt to interact with general fear and health concerns around COVID-19 to further delay care.

“we spend a lot of time encouraging them to have such a low threshold to come in, but then it really became very muddy, that time of COVID [first wave], because they didn't know what was more dangerous, coming to the hospital or staying at home, you know? So I think we did find some late presentations…that was a bit of a worry really.” Medical 115

Additionally, staff struggled with the intersection of increased need but decreased opportunities to engage with women and provide care:

“the higher incidents of DV as well as the challenges of actual engagement with clients…typically I would invite people in…they were typically coming for their antenatal appointments anyway…but there was a drop off…because they just didn't really want to be coming in accessing healthcare…I think it's really harmful to not deliver a service to clients that need it.” Allied Health 114.

Woman-centered care considers the woman's individual circumstances and aims to meet the woman's social, emotional, physical, psychological, spiritual, and cultural needs (18). The women expressed to the midwives a lack of being recognized in the changed processes:

“Just from the feedback that we've had from women, was that they did find it quite dehumanizing…one woman said to us that she'd lost…that sort of dream of a pregnancy that she'd had. She said that's been taken away from me because I've had no face-to-face visits; I haven't really been able to enjoy this pregnancy.” Midwife 105

“I was noticing a high level of anxiety amongst the women, and many, many women said things to me like ‘Oh, now for the first time I feel cared about' and they were 28 weeks pregnant.” Midwife 109

This loss of ability for women and health professionals to work in partnership affected choices, communication, and education that may have both short- and long-term health impacts for women and families post-pandemic.

#### Changes to Delivery of Care—Effect on Staff

Most staff reported negative perceptions of the change in care. The lack of physical contact and additional barriers to care of personal protective equipment, especially mask wearing, were seen to have impact on communication and ability to engage with women as these midwives describe:

“Wearing the masks has made it quite impersonal.” Midwife 111

“And when she's got the mask on, I've got a mask on, it's so hard to get the subtle emotion and even to show my emotion to her as well.” Midwife 110

At the same time, staff were struggling with the increased workload from changed care processes in conjunction with increased sick leave due to need to be vigilant around COVID-19 symptoms.

“There is definitely a lot more tasks and…people were taking more sick leave because they had to get swabs whereas [pre-COVID] they probably just would've come to work.” Midwife 101

Staff also perceived this as negatively affecting continuity of care for women:

“We've had more sickness with staff because you can't just come in with a runny nose anymore…so we've had less staff coming in to do their clinics which means that women get less continuity.” Midwife 104

On the positive side, some staff noted that although they felt telehealth had a limited role in the maternity setting for patient care, online staff meetings were quite beneficial:

“one thing I think is great is…online applications for communication. I love them in terms of meetings…it's very economical from a time perspective.” Midwife 109

Staff had to cope with changed practices in how they delivered their care, constantly changing restrictions and requirements while coping with the impact of the pandemic personally and in their workplace:

“on some days it was hour by hour things were changing…it was a significant workload…we all burned out by the end of last year [2020].” Midwife 101

## Discussion

Our mixed-methods study of three metropolitan Sydney hospitals providing maternity care found that staff perceived a major and mostly negative impact of the COVID-19 pandemic on both pregnancy care in general, and more specifically regarding screening and care for DFV and mental health issues. Although the focus of this study was primarily on pandemic impacts on psychosocial screening in pregnancy care, the interviews in particular uncovered broader themes of the overall changes to delivery of care, including telehealth. The pandemic created an immediate impact requiring changes to established delivery of models of maternity care. Women-centered care offering choice, control, and continuity was removed for both woman and the health professionals. New rapidly changing ways of working including a change to telehealth were not formally evaluated in established in maternity services or midwifery continuity of models of care, but imposed by health services as a part of pandemic response and restrictions (12). Staff had particular concern around negative impact on whether DFV screening was performed, its timeliness, and the equity of screening telehealth was acknowledged as having both positive and negative aspects, with perceived positive aspects including convenience and reduced travel time for women. Negative aspects included inability to perform physical examination, difficulty picking up non-verbal cues, issues for women requiring interpreters, and safety of asking certain questions, e.g., regarding DFV.

Changes to delivery of care saw staff express that although some telehealth would be a useful tool going forward for pregnancy care, this should still be a minority of visits and that those who were high-risk either medically or psychosocially were not suitable for any telehealth. Regarding restrictions as part of face-to-face care (mask-wearing and visitor restrictions), interviewees felt overall relatively positive about visitor restrictions, particularly for providing better immediate postnatal care. However, mask-wearing/personal protective equipment use and lack of physical contact with women were seen as further diminishing qualities of care and the major negative impacts on the personal nature of maternity care delivery. Staff were clearly concerned about the difficulties of providing woman-centered care, a central tenet of Australian pregnancy care guidelines, (18) and articulated the moral hazard of balancing perceived community needs against providing appropriate care. This echoes findings among the broader DFV Australian workforce, who have experienced increased workload but also its unrelenting, exhausting nature in addition for concern for the future: “it's who we're not seeing that worries me” (19). Thus, as well as for women and their families, the short- and long-term impacts for staff post-pandemic need to be considered.

In the Australian context, a number of qualitative studies have been performed to date from the perspective of midwives regarding provision of maternity care (20–22) and of women regarding their maternity care experiences (23, 24). These largely align with the more general findings of this study, with midwives reporting challenges to provision of woman-centered care (20, 22), and difficulties coping with rapid changes to care (including telehealth) and “COVID-19 causing chaos” (20–22). As in this study, “silver linings” included the perceived positive impact of visiting restrictions on postnatal care (20, 22). The women themselves noted the impact of navigating a changing healthcare system, the impact on preparedness in pregnancy and for parenting, and facing the uncertainty of a pandemic (23, 24).

Regarding mental health and/or DFV specifically, our findings suggest that changes in maternity care delivery in Australia, with face-to-face visits later in pregnancy and fewer in number, are likely to compound the perinatal mental health and DFV impact of the pandemic, through delay in screening and care, and in some cases missed screening altogether. This issue was also noted by Hearn et al. in their study of midwives in Melbourne, Australia, who repeatedly voiced concerns around screening for family violence and noting how unsafe this was to do by telephone (21). Our findings also suggest a disparity in screening and care impacts for women from diverse backgrounds, as the unit with the highest proportion of overseas-born and non-English speaking women had a significantly higher staff perception of negative impact of the pandemic on DFV and mental health screening and care. This is also reflected in the fact that almost two times as many surveyed staff felt that telehealth increases inequities in pregnancy care vs. decreases it. This has profound implications for ensuring ongoing and timely access to DFV screening and services as part of high quality, safe, and equitable pregnancy care. Staff further explained that regarding DFV and mental health screening, deferral of screening until the woman could be seen face-to-face then resulted in delayed care for affected women and potentially missed screening and care altogether. Any increased incidence of stress and mental health disorders during the pandemic was therefore not adequately addressed (25).

### Strengths and Limitations of the Study

The strengths of the study include its mixed-methods design, allowing for both breadth and depth of perception of maternity care providers. However, the decision to produce (in the interests of timely study completion) the interview guide and survey questions in parallel, rather than using survey findings to inform the interview questions, is a potential limitation. Another strength is that although many findings related to general insights on staff regarding pregnancy care, the study's focus on the specific but extremely common issue of psychosocial screening and care during pregnancy also allowed for a deeper understanding of COVID-19 pandemic impacts in this area than would have been achieved by only a general exploration of maternity effects. Inclusion of the perspectives of allied health and medical maternity healthcare professionals in addition to those of midwives contributed to a more holistic view of maternity service provision in this area. Limitations include the geographical limitation of the work to three hospitals in a specific area of Sydney: although these hospitals do service a range of pregnant populations from low-risk through to very high risk, and with differing sociodemographic catchments, experiences of staff at these hospitals may not be the representative of the broader Australian maternity system. The lack of involvement of the women themselves also limits the conclusions that can be drawn.

### Implications for Practice and Conclusion

Our study findings suggest that the adaptations to SESLHD maternity care due to the pandemic, particularly increased telehealth and reduced face-to-face visits, have some ongoing utilities for selected women and at appropriate times during the pregnancy. However, the pandemic practice of switching the “booking-in” visit to telehealth should revert to face-to-face, both to ensure that safe and appropriate psychosocial screening occurs in the first half of pregnancy, so that services for those experiencing mental health and/or DFV issues can be instituted with sufficient time to have a positive impact prior to birth. Staff perceive follow-up visits *via* telehealth to be most appropriate for those with low-risk pregnancies, who have already had children, and who live further away from the hospital. Women at high risk either because of physical health or due to mental health, DFV, and/or other social concerns are unsuitable to be seen *via* telehealth going forward. Other pandemic restrictions perceived to be positive by staff for either general care (partner only on postnatal ward/no other visitors allowed) or psychosocial/DFV screening (limited partners at antenatal visits) are not broadly sustainable on an ongoing basis. However, more restricted general (vs. partner only) visiting hours on postnatal wards may be able to be implemented to sustain postnatal care improvements.

## Data Availability Statement

The raw data supporting the conclusions of this article will be made available by the authors, on reasonable request.

## Ethics Statement

The studies involving human participants were reviewed and approved by South-Eastern Sydney Local Health District Human Research Ethics Committee. The patients/participants provided their written informed consent to participate in this study.

## Author Contributions

AH, LE, LR, AL, and PC: study concept and design. SG, AH, LE, LR, and AL: data acquisition. JY, SG, AH, and LE: data analysis. AH, JY, SG, LR, AL, JS, PC, and LE: data interpretation and manuscript editing. AH: manuscript drafting. All authors have approved the final submitted manuscript.

## Funding

This research was supported by a UNSW Research COVID-19: Rapid Response Research Fund philanthropic grant. The funders had no input into the design or conduct of the research project, manuscript content or decision to publish. Patricia Cullen is funded by a NHMRC Early Career Fellowship (Grant ID: APP1158223).

## Conflict of Interest

The authors declare that the research was conducted in the absence of any commercial or financial relationships that could be construed as a potential conflict of interest.

## Publisher's Note

All claims expressed in this article are solely those of the authors and do not necessarily represent those of their affiliated organizations, or those of the publisher, the editors and the reviewers. Any product that may be evaluated in this article, or claim that may be made by its manufacturer, is not guaranteed or endorsed by the publisher.
