# Supplementary material for: Effects of the COVID-19 Pandemic and Telehealth on Antenatal Screening and Services, Including for Mental Health and Domestic Violence: An Australian Mixed-Methods Study
Source: Front Glob Womens Health. 2022 Jun 22;3:819953. doi: 10.3389/fgwh.2022.819953 (PMC9257034; doi:10.3389/fgwh.2022.819953)
Supplement: Supplementary file 1 [file Data_Sheet_1.ZIP › Supplementary Material 1.docx]

**Supplementary Material 1. Health Care Practitioner Survey**

Part 1. Survey Introduction and participant information sheet

During the COVID-19 pandemic maternity services are experiencing many changes in clinical practices and workplace environments. Many visits have been changed to online, including in some SESLHD centres the “booking-in” pregnancy visit, which is usually one of the time-points for routine psychosocial screening. The study researchers are trying to ascertain the effects on the COVID-19 pandemic on maternity care in SESLHD, particularly on psychosocial screening (including mental health and domestic violence screening), on pregnancy care more generally, and on pregnancy outcomes. As part of the research we are conducting a survey of maternity healthcare workers at the Royal Hospital for Women, St George Hospital and Sutherland Hospital to find out their views on how the COVID-19 pandemic has affected pregnancy care.

If you are: 1) a health care worker working with pregnant mothers in SESLHD during the pandemic in 2020, *and* 2) you were also working in SESLHD maternity services pre-pandemic (during 2019 or earlier), you are invited to take part in this survey to help ascertain the effects of the COVID-19 pandemic on provision of maternity care in SESLHD.

The study is being undertaken by Dr Amanda Henry and Dr Lynne Roberts from the Department of Women’s and Children’s Health, St George Hospital and the University of New South Wales, Clinical A/Prof Karen Walker from The George Institute for Global Health, and Anne Lainchbury and Virgina Speer from the Royal Hospital for Women. This study is being supported by a grant from the University of NSW Rapid Response COVID-19 fund. Ethics approval has been obtained from the SESLHD ethics committee. (HREC xxxx).

The survey contains some questions about you (e.g. your area of pregnancy care), and then some questions about your perceptions of the impact of COVID-19 on pregnancy care provision. The survey should take between 10 and 15 minutes to complete, depending on your responses. You can stop this survey at any time and choose not to submit your responses. All data collected is anonymous and there is no way you can be identified. It is intended to present and publish this data, however all data will be presented in groups (e.g. midwife responses versus doctor responses versus allied health responses), not as individual responses.

If you are willing to participate in the survey, please tick this box as an acknowledgment that you have read the participant information, and that you consent to participate in the survey. This will then open the survey.

❒ I acknowledge that I have read the participant information and that I consent to participate in the survey.

Part 2 Survey Questions

About you (For each question, please indicate the single most correct option).

Discipline/role:

- Midwife

o Primary area (please choose single most correct answer):

- Antenatal care
- Postnatal care
- Intrapartum care
- Midwifery Group Practice
- CMC, CMS or CME
- Management
- All areas
- Midwife, prefer not to say area
- Medical

o Primary role (please choose single most correct answer):

- Obstetrician, work predominantly public
- Obstetrician, work equal public and private
- Obstetrician, work predominantly private
- Obstetric Registrar/Resident
- Medical, prefer not to say type
- Allied health

o Primary role (please choose single most correct answer):

- Social work
- Physiotherapy
- Nutrition/dietetics
- Genetics
- Mental Health
- Other
- Allied health, Prefer not to say area
- Prefer not to say

My sex:

- Female
- Male
- Non-binary
- Prefer not to say

My age:

- <25
- 25-34
- 35-44
- 45-54
- 55 and older
- Prefer not to say

My years of experience in pregnancy care:

- 5 or less
- 6 to 10
- Between 11 and 15
- 16 or more
- Prefer not to say

My primary affiliated public hospital:

- Royal Hospital for Women
- St George Hospital
- Sutherland Hospital
- Prefer not to say

Perceptions as a maternity care provider of the impact of the COVID-19 pandemic on the provision of pregnancy care

From your perspective, what do you feel has been the overall impact of COVID-19 on the *delivery* of pregnancy care? (e.g. ensuring women get required visits)

- Extremely negative
- Somewhat negative
- Neutral/neither negative nor positive
- Somewhat positive
- Extremely positive
- Not sure

From your perspective, what do you feel has been the overall impact of COVID-19 on the *delivery* of screening for domestic and family violence as part of pregnancy care? (i.e. on whether domestic violence screening is occurring or not as part of routine pregnancy care).

- Extremely negative
- Somewhat negative
- Neutral/neither negative nor positive
- Somewhat positive
- Extremely positive
- Not sure

From your perspective, what do you feel has been the overall impact of COVID-19 on the *delivery* of screening for mental health disorders as part of pregnancy care? (e.g. performance of Edinburgh Depression score/asking regarding mental health history at time of booking).

- Extremely negative
- Somewhat negative
- Neutral/neither negative nor positive
- Somewhat positive
- Extremely positive
- Not sure

From your perspective, what do you feel has been the overall impact of COVID-19 on the *timeliness* of pregnancy care? (e.g. ensuring women are booked in to hospital by the end of the first trimester, frequency of visits)

- Extremely negative
- Somewhat negative
- Neutral/neither negative nor positive
- Somewhat positive
- Extremely positive
- Not sure

From your perspective, what do you feel has been the overall impact of COVID-19 on the *timeliness* of screening for domestic and family violence as part of pregnancy care? (i.e. on whether domestic violence screening is occurring early in pregnancy).

- Extremely negative
- Somewhat negative
- Neutral/neither negative nor positive
- Somewhat positive
- Extremely positive
- Not sure

From your perspective, what do you feel has been the overall impact of COVID-19 on the *timeliness* of screening for mental health disorders as part of pregnancy care? (e.g. performance of Edinburgh Depression score/asking regarding mental health history early in pregnancy).

- Extremely negative
- Somewhat negative
- Neutral/neither negative nor positive
- Somewhat positive
- Extremely positive
- Not sure

From your perspective, what do you feel has been the overall impact of COVID-19 on the *quality* of pregnancy care? (e.g. ensuring women receive all the information and support that you would normally expect to be provided as part of overall pregnancy care)

- Extremely negative
- Somewhat negative
- Neutral/neither negative nor positive
- Somewhat positive
- Extremely positive
- Not sure

From your perspective, what do you feel has been the overall impact of COVID-19 on the *quality* of screening *and care* regarding domestic and family violence as part of pregnancy care? (e.g. being able to give the appropriate support and safety interventions if a disclosure of domestic violence is made).

- Extremely negative
- Somewhat negative
- Neutral/neither negative nor positive
- Somewhat positive
- Extremely positive
- Not sure

From your perspective, what do you feel has been the overall impact of COVID-19 on the *quality* of screening *and care* for mental health disorders as part of pregnancy care? (e.g. quality follow-up services and support if a high Edinburgh Perinatal Depression Score is found).

- Extremely negative
- Somewhat negative
- Neutral/neither negative nor positive
- Somewhat positive
- Extremely positive
- Not sure

A rapid shift to providing some antenatal visits by telehealth has been one of the features of the COVID-19 pandemic. The following questions explore your perceptions, as a provider of maternity care services, of the impacts of telehealth on antenatal care.

In 2019 i.e. pre-pandemic, what proportion of antenatal visits (or pregnancy care services that your department provides, if you are an allied health worker) would have been provided by telehealth?

- None
- Occasional (<10%)
- Sometimes (10-25%)
- Often (26-50%)
- Majority (over 50%)
- Not sure/couldn’t say

*During* the pandemic i.e. from March 2020, what proportion of antenatal visits (or pregnancy care services that your department provides, if you are an allied health worker) do you estimate has been provided by telehealth?

- None (0%)
- Occasional (<10%)
- Sometimes (10-25%)
- Often (26-50%)
- Majority (over 50%)
- Not sure/couldn’t say

What do you see as the positive features of telehealth as part of pregnancy care? (in general, not just specifically due to COVID-19). Please select all answers that apply.

- No positive features from my perspective
- Convenient for woman
- Convenient for staff
- Helps with scheduling
- Reduces overcrowding in clinics
- Useful to reduce long travelling times to hospital for some women
- Takes less time than face to face visit
- Decreases inequities in delivery of pregnancy care
- Other, please state …………………………………………………………………………

What do you see as the negative features of telehealth as part of pregnancy care? (in general, not just specifically due to COVID-19). Please select all answers that apply.

- No negative features from my perspective
- Can’t do physical examination
- Difficult to pick up on non-verbal cues from woman
- Awkward asking some questions (please state which topics …………………………..)
- Unsure if safe to ask some questions (please state which topics ……………………)
- Difficult if interpreter required
- Takes longer than face to face visit
- Increases inequities in delivery of pregnancy care
- Other, please state ………………………………………………..

Post-pandemic, do you think that telehealth should continue for some aspects of pregnancy care/some antenatal visits?

- Yes, definitely (about what proportion of visits? XX%)
- Yes, probably (about what proportion of visits? XX%)
- Neutral, unsure
- No, probably not
- No, definitely not

Why or why not? ………………………………………………………………………………………………………………………………..

Are there any groups of pregnant women who you think are particularly suited to having at least some visits by telehealth? (e.g. multiparous, low risk of complications)

- Yes. Please state which groups ……………………………………………………………….
- Neutral, unsure
- No

Are there any groups of pregnant women who you think are NOT suitable for having at least some visits by telehealth? (e.g. non-English speaking background, past history of complications)

- Yes. Please state which groups ……………………………………………………………….
- Neutral, unsure
- No

Another impact of the pandemic has been on specific pregnancy care groups/initiatives e.g. centering groups, vaginal birth after Caesarean education sessions, Young Mums. In your maternity service, what do you feel has been the overall impact on the *provision* of pregnancy care and education groups?

- No impact/all still going ahead
- Some impact (up to half of groups cancelled or changed to online)
- Major impact (half or more of groups cancelled or changed to online)
- Not sure/don’t know

From your perspective, what impact would permanently changing pregnancy care and education groups to either telehealth/online or a hybrid of face to face and telehealth have?

- Extremely negative
- Somewhat negative
- Neutral/neither negative nor positive
- Somewhat positive
- Extremely positive
- Not sure

Comments……………………………………………………………

If you have any general comments or additional perspectives about the provision of pregnancy care pre-pandemic versus during pandemic, particularly as regards psychosocial screening and care, please let us know.

…………………………………………………………………………………………………………………………………………………………

Thank you very much for completing this survey. If you would be interested in being interviewed about your experiences of working in maternity care during the COVID-19 pandemic, please click on the box below and you will be directed to another page to fill in your contact details. This page will be kept separate from your survey responses to maintain the anonymity of your survey responses.

(LINK TO GIVE DETAILS ABOUT INTERVIEW IF INTERESTED).
