# Supplementary material for: Effects of the COVID-19 Pandemic and Telehealth on Antenatal Screening and Services, Including for Mental Health and Domestic Violence: An Australian Mixed-Methods Study
Source: Front Glob Womens Health. 2022 Jun 22;3:819953. doi: 10.3389/fgwh.2022.819953 (PMC9257034; doi:10.3389/fgwh.2022.819953)
Supplement: Supplementary file 1 [file Data_Sheet_1.ZIP › Supplementary Material 2.docx]

**Supplementary Material 2. Qualitative interview guide**
