# Supplementary material for: Effects of the COVID-19 Pandemic and Telehealth on Antenatal Screening and Services, Including for Mental Health and Domestic Violence: An Australian Mixed-Methods Study
Source: Front Glob Womens Health. 2022 Jun 22;3:819953. doi: 10.3389/fgwh.2022.819953 (PMC9257034; doi:10.3389/fgwh.2022.819953)
Supplement: Supplementary file 1 [file Data_Sheet_1.ZIP › Supplementary Material 3.docx]

**Supplementary Material 3. Impact of COVID-19 on maternal service provision and outcomes**

**Distress and Safety Protocol for the participants**

Background

Some of the intervew questions regarding impact of COVID-19 on maternal service provisions and outcomes may cause distress for participants (maternity staff), as the interview includes discussion of mental health screening and domestic and family violence screening and management during pregnancy. This could be the case either because discussion of these topics triggers distress experienced by the maternity staff member when assisting with a previous case of domestic violence or mental health issues during the course of her/his work, and/or through past personal trauma of the staff member relating to these issues. All study staff need to be vigilant for signs of distress in study participants.

The following protocol will be put in place should a participant became distressed and require either additional or ongoing assistance. A range of services could be offered depending on her circumstances.

Protocol

Prior to the commencement of patient questionnaires/interview, information regarding the counselling available should it be required will be provided to all study participants by the research staff. Research staff will provide sufficient information regarding the risks and benefits of the research so that individuals may freely accept or decline participation. This information will be made available to the participant prior to and questionnaires/interview commencing. An additional notification of this information will also be given to those participants should they become distressed during the actual study.

**Strategies to assist those distressed during questionnaires/interview**

Should a participant become uncomfortable or distressed while completing any questions, the following actions will be taken by the interviewer/study staff:

1. Suggest that it is appropriate that the questionnaires/interview be terminated.

2. If the participant wishes this to happen, the interview will be ceased.

3. In the case where a counsellor or GP is not readily accessible a member of the research team who is a health professional (which will usually be the researcher performing the interviews) will spend time with the participant and provide assistance, within the scope of their abilities, to discuss their concerns and support them.

4. After seeking advice from the Chief investigator, a recommendation will be made that the participant speak to a counselling professional to discuss their concerns and be referred if they agree.

- 1. • If the participant has a general practitioner (GP) involved in her care it may be more appropriate to refer her to her GP who is already familiar with their history and would provide continuity of care. In this case the options of a counsellor would be provided to the participants as well. (Medicare will cover 10 sessions of counselling annually).

5. The intended outcome of the activation of this protocol will be a comprehensive assessment, and presentation of options to the woman regarding ongoing counselling or other management as appropriate.

6. A follow-up phone call will be made by the interviewer the following day to ensure that the participant is well and to determine feasibility of a follow up interview if one is planned.

**Distress and safety Protocol: Researchers**

Although study staff are experienced in perinatal research including complicated psychosocial care, the possibility of staff members becoming distressed by the related experiences of the participants (maternity staff) should not be discounted. The following protocol will be put in place should a researcher become distressed or be at risk during their work with the study and require emergency, additional or ongoing assistance. A range of services could be offered depending on the circumstances.

**Strategies to assist distressed research staff**

1. The research staff have regular meetings with their supervisors

2. The researcher will be referred to a counselling professional to discuss their concerns and/or a referral made to their Employee Assistance Program

3. The researcher will always carry a mobile phone while working in the field and will share the contact details and location of the interviews with research team

**Conclusion**

It is the researcher’s duty of care to ensure that there is a balanced consideration of the benefits against the risks. The research will ensure these strategies are put in place prior to commencing the interviews or discussions.

Below is the step by step guided protocol adapted from Draucker C B, Martsolf D S and Poole C (2009) Developing Distress Protocols for research on Sensitive Topics. *Archives of Psychiatric Nursing* 23 (5) pp 343-350

**Participants’** **Safety & Distress Protocol**:
